# Coarse-Grained Neural Network Model of the Basal Ganglia to Simulate Reinforcement Learning Tasks

**DOI:** 10.3390/brainsci12020262

**Published:** 2022-02-14

**Authors:** Jarosław Drapała, Dorota Frydecka

**Affiliations:** 1 Department of Computer Science and Systems Engineering, Faculty of Information and Communication Technology, Wroclaw University of Science and Technology, Wybrzeze Wyspianskiego Street 27, 50-370 Wroclaw, Poland; 2 Department of Psychiatry, Wroclaw Medical University, Pasteur Street 10, 50-367 Wroclaw, Poland; dfrydecka@gmail.com

**Keywords:** neural network modeling, basal ganglia, reinforcement learning, probabilistic selection task, probabilistic reversal learning task, instructional probabilistic selection task

## Abstract

Computational models of the basal ganglia (BG) provide a mechanistic account of different phenomena observed during reinforcement learning tasks performed by healthy individuals, as well as by patients with various nervous or mental disorders. The aim of the present work was to develop a BG model that could represent a good compromise between simplicity and completeness. Based on more complex (fine-grained neural network, FGNN) models, we developed a new (coarse-grained neural network, CGNN) model by replacing layers of neurons with single nodes that represent the collective behavior of a given layer while preserving the fundamental anatomical structures of BG. We then compared the functionality of both the FGNN and CGNN models with respect to several reinforcement learning tasks that are based on BG circuitry, such as the Probabilistic Selection Task, Probabilistic Reversal Learning Task and Instructed Probabilistic Selection Task. We showed that CGNN still has a functionality that mirrors the behavior of the most often used reinforcement learning tasks in human studies. The simplification of the CGNN model reduces its flexibility but improves the readability of the signal flow in comparison to more detailed FGNN models and, thus, can help to a greater extent in the translation between clinical neuroscience and computational modeling.

## 1. Introduction

The basal ganglia (BG) are a set of subcortical nuclei responsible primarily for motor control [1]; however, they also play roles in motor learning, executive functions, emotional processing and action inhibition. The BG have been wildly researched within the framework of cognitive neuroscience in an attempt to gain a deeper understanding of the neuronal basis of psychiatric and neurological disorders [2]. Many computational models have been proposed by researchers with the aim of studying the internal structure and functioning of the BG and providing deeper insight into the outcomes of experiments involving human subjects [3,4]. Accumulating evidence suggests that the contribution of various BG components may be described within a reinforcement learning model [5]. The BG selects from the available actions represented in the cortex, and it triggers the execution of one selected action while suppressing other actions [6]. The BG functions as a generic action selection system that uses the feedback mechanism to improve its own performance [7]. The reinforcement signal, referred to as a reward prediction error signal, is transferred from the substantia nigra pars compacta (SNc) and the ventral tegmental area (VTA) to the BG through the dopaminergic neurons that fire in proportion to the difference between the expected and actual reward [8]. The error signal from midbrain dopaminergic neurons, together with environmental cues from the cortex, create convergent information that modifies the activity of the striatum [9]. The dynamic modulation of striatal activity is causally related to behavioral changes [10].

The main input of the BG is the striatum, which receives information from the cortex and thalamus via glutaminergic neurons, as well as dopaminergic projections from SNc [11]. The dopamine signal produced by these connections provides the BG with information about the performance of the task by means of the reward prediction error, a key signal in many reinforcement learning models [8,12,13]. The prediction error signals the difference between the observed and expected outcomes: a positive prediction error signals that the outcome was better than expected, while a negative prediction error signals that the outcome was worse than expected [14,15]. The magnitude of phasic dopamine bursts represents a positive prediction error, while, in the case of a negative prediction error, the dopamine level is reduced. In neurons with dopaminergic D1 receptors, a rise in the dopamine level produces long-term potentiation (LTP), while, in neurons expressing dopaminergic D2 receptors, it produces long-term depression LPD [16]. On the other hand, a reduced level of dopamine produces LPD in the D1 receptors and LTP in the D2 receptors. There are two pathways, known as the direct pathway and indirect pathway. The direct pathway comprises striatal D1 cells, and it is directly connected to the globus pallidus pars interna (GPi), while the indirect pathway includes D2 cells and is connected to the globus pallidus pars externa (GPe). The direct pathway reduces the tonic activity of GPi and, thus, reduces the level of inhibition from GPi to the thalamus. The indirect pathway, through the inhibitory connections between D2 cells of the striatum and GPe, removes the continuous inhibition provided by the tonic firing from GPe to GPi. The direct pathway is associated with GO functions, while the indirect pathway with NoGo functions, such as inhibiting the incorrect actions [17,18].

In recent years, there have been several models of BG proposed to allow the simulation of behavioral experiments [19,20,21]. The BG models differ with respect to many variables, such as learning algorithms, structure interconnectedness and the level of complexity. The simplest approach is to assume that a single population of neurons represents one value that changes in response to the input signals [22]. The output of a single artificial neuron is commonly interpreted as a mean firing rate of the whole group of neural cells [23]. On the other hand, there are models that try to represent the population of neurons in full detail, using large pools of spiking neuron models [24,25,26]. The most complete and detailed model of a single neuron is the Hodgkin–Huxley model, which implementation requires a very high level of computational resources [27]. Neuroscientists have developed several biologically plausible simplifications, such as the leaky integrate-and-fire model, the model proposed by Izhikkievich and many others (presented in References [28,29]). Some of the models use variations of Hebbian and competitive learning [25], while others modeling spiking neural networks implement the spike-timing-dependent plasticity (STDP) algorithm [28,30]. In some cases, scientists have proposed a tailormade learning algorithm derived from their line of research [19]. The wide selection and availability of modeling frameworks make it relatively easy to develop more and more complex models of the BG by adding additional elements with an aim to increase the functionality of the model [31,32,33,34,35]. However, the fundamental principles behind the model design lose their explanatory power with every adjustable element added to the model [36]. A typical problem with detailed and complex models containing many neuronal structures and/or many parameters is the issue of overfitting [37]. A model with too little capacity cannot learn the given problem, whereas a model with too much capacity can learn it too well and overfit the training dataset; thus, such a model does not have the ability to generalize the knowledge well [38]. Thus, the selection of a model size that maximizes generalization is an important topic that has been given a lot of attention in the last years as the field of cognitive neuroscience develops [39].

The aim of the present work was to develop a BG model that could represent a good compromise between simplicity and completeness. Our starting point was the BG model proposed by Frank et al. [19] that has been used in a wide scope of research on healthy volunteers, as well as on people with neurological disorders (mainly Parkinson’s disease) [22] and psychiatric conditions (mainly schizophrenia and attention deficit/hyperactivity disorder) [32,40]. We refer to this network as the fine-grained neural network (FGNN), and based on its structure, we developed a new coarse-grained neural network (CGNN) replacing layers of neurons with single nodes that represent the collective behavior of the given layer. We then compared the functionality of both the FGNN and CGNN models with respect to several reinforcement learning tasks that are based on the BG circuitry, such as the Probabilistic Selection Task [22,41], Probabilistic Reversal Learning Task [42] and Instructed Probabilistic Selection Task [43,44], to assess whether a more simple approach in modeling can produce similar results.

## 2. Methods and Models

### 2.1. Fine-Grained Neural Neetwork (FGNN) Model of Basal Ganglia

**Figure 1:**
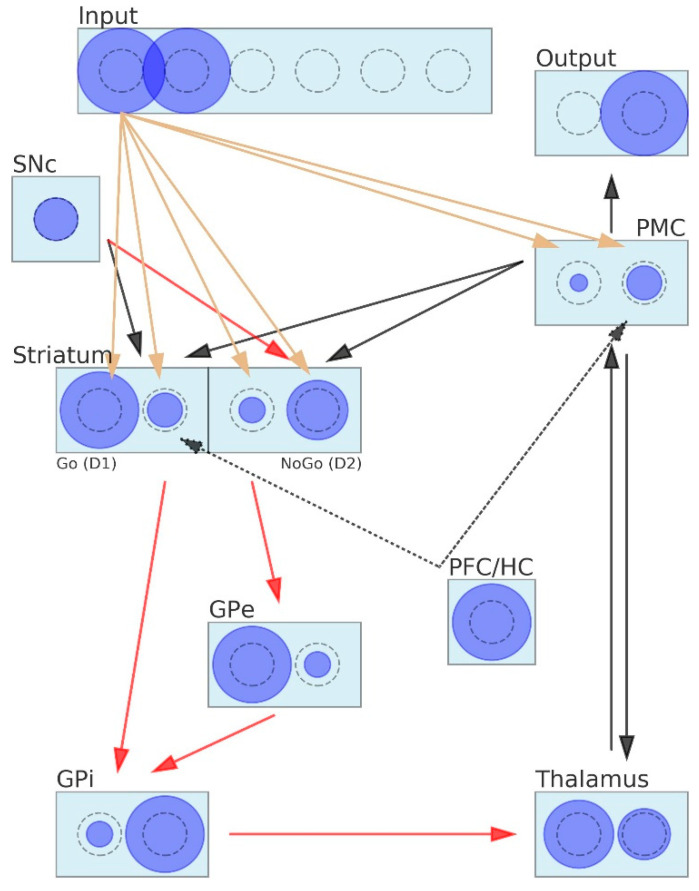
The fine-grained neural network (FGNN) model of the basal ganglia (BG), including the direct Go) and indirect NoGo pathways. The Go elements disinhibit the thalamus via GPi, thereby facilitating the execution of an action represented in the cortex. The NoGo elements suppress actions from being executed by increasing the inhibition of the thalamus. Dopamine from the SNc projected to the striatum causes the excitation of Go cells via the D1 receptors and inhibition of NoGo via the D2 receptors. Squares represent units, with circles reflecting neural activity. Arrows represent connections between layers or neurons: red for inhibitory and other colors for excitatory, olive for modifiable connections and other colors for fixed connections. Abbreviations: SNc—substantia nigra pars compacta, PFC/HC—prefrontal cortex/hippocampus, GPi and GPe—globus pallidus pars interna and pars externa and PMC—premotor cortex.

The general view of the neural network representing the BG and associated structures is presented in Figure 1. The BG circuitry includes the following layers: input, output, premotor cortex (PMC), striatum, substantia nigra pars compacta (SNc), globus pallidus pars externa and interna (GPe and GPi), thalamus and prefrontal cortex/hippocampus (PFC/HC). Rectangles represent the nuclei (layer) and circles the populations of neurons. Further on, we will follow the convention by which the whole population of neighboring neurons expressing a joint activity is collectively referred to as a neuron. The diameter of the circles is proportional to the average firing rate of a neuron (or, simply, the neuron firing rate). Grey dashed circles indicate half the scale of the neuronal activity, meaning that the neuron of exactly this size is firing at the baseline rate.

Arrows represent connections between layers or neurons: red for inhibitory and other colors for excitatory, olive for modifiable connections and other colors for fixed connections. This means that, in the proposed model, learning takes place only between the input layer and the striatum and between the input and the PMC. When an arrowhead goes inside the neuron area, this indicates a one-to-one connection between a couple of neurons belonging to different layers. When the arrowhead ends before the rectangle area, we are dealing with dense (all-to-all) connections or a series of parallel connections between corresponding neurons belonging to connected layers. All the red arrows and arrows connecting the SNc with the striatum stand for parallel types of connections. In order to keep the network diagram readable, we drew out only connections between one selected neuron of the input layer and the striatum. Drawing all input–striatum and input–PMC interconnections would make the diagram obscure. The width of the olive arrow is proportional to the strength of the synaptic connection between neurons and may vary as the learning proceeds. The black arrows originating in the PFC/HC are dashed to indicate that the PFC/HC nuclei and associated connections are present only when simulating selection tasks with instructions. The striatum is divided into two separate layers: D1 containing Go neurons and D2 composed of NoGo neurons.

From a black box perspective, the BG receives signals from the sensory and motor parts of the cortex and outputs signals to the PMC via the thalamus. Signals flow through the network in the following way. The input layer encodes stimuli, assuming that each stimulus is represented by the activity of one particular neuron. For instance, in Figure 1, the input layer is prepared to encode three pairs of stimuli, and in the current trial, the first pair of stimuli is presented to the network. The input layer projects to the striatum, activating the Go and NoGo units, and then, a series of inhibitory connections follows. Go neurons inhibit the corresponding GPi units, which further inhibit the corresponding neurons of the thalamus layer. The signals generated by the NoGo neurons pass through the double inhibitory connections with the GPe layer in between, resulting in the additional inhibition of the GPi. Note, however, that the logic behind the double inhibitory action makes the NoGo neurons to disinhibit the corresponding units of the GPi. The tonic activity of the GPi inhibits the thalamus, unless some unit of the GPi becomes muted and, in consequence, the corresponding neuron of the thalamus becomes unlocked. The thalamus serves as a gating mechanism for the PMC facilitating the execution of one particular action, which is performed during two-way communication between those layers. The output layer of the network represents the final decision made by the network, and we may think of it as a motor command resulting in the choice of the stimulus on the left or on the right (e.g., pushing the appropriate button). Motor commands may be partially evoked by signals sent directly from the input layer due to the input–PMC projections. The synaptic weights of those connections are assumed to be modifiable.

Striatal Go/NoGo representations are learned via phasic changes in simulated dopamine firing in the SNc compacta layer during positive and negative reinforcement. The SNc is involved in releasing the neurotransmitter dopamine (DA) in response to events of unexpected reward. Positive rewards produce DA bursts (high peaks of the DA level), and negative rewards lead to DA dips (the DA level falls deep below the baseline). DA bursts make the Go neurons more active and NoGo neurons less active. Conversely, DA dips inhibit the Go neurons and excite the NoGo neurons. To fully close this feedback loop, PMC–striatum signaling is needed to recall which motor action led to the reward and to indicate the striatum neural units that must be exposed to DA action. The PFC/HC role is to incorporate instructions into the learning process. There are two ways the PFC/HC may lean the BG towards the instructed stimulus. It may bias the striatum, where the value of actions is learned (distorting the learning process) or it may affect the behavior by modulating the response of the PMC (overriding the learning process). It is very likely that both processes take place simultaneously.

### 2.2. Coarse-Grained Neural Neetwork (CGNN) Model of Basal Ganglia

#### 2.2.1. Activity of Neurons and Connectivity between Neurons in the CGNN

Here, we describe the activity of neurons and their connectivity in the CGNN model of the BG.

1. The SNc is represented by a single neuron that can return one of the following three values: dopamine dip = 0, tonic dopamine = 0.5 or dopamine burst = 1;
2. The input layer encodes stimuli. Each stimulus is represented by one neuron that returns “1” if its stimulus appears on the screen and “0” otherwise;
3. Similar rules apply to the output layer, where each decision is encoded by one neuron. Since only one decision may be made at the moment, only one neuron is allowed to return 1, whereas all others are inhibited and return 0;
4. The PFC/HC is represented by a single neuron that passes 1 to that neuron of the striatum that is associated with the instructed stimulus. The PFC/HC is active in the instructed probabilistic selection task;
5. Neurons belonging to the layers PMC, striatum, GPe, GPi and thalamus may return values belonging to the interval <0,1>;
6. The number of neurons included in the layers Input, Output, PMC, GPe, GPi and thalamus is equal to the total number of stimuli in the learning task;
7. The striatum layer contains twice as many neurons as the total number of stimuli in the learning task. This layer is composed of two subparts: D1 (including neurons representing Go signals) and D2 (including neurons representing NoGo signals);
8. The PFC/HC is connected to those D1 and the PMC neurons that stand for the instructed stimulus;
9. The activity of the striatum neurons is evaluated according to the following formula (note that the last term of the equation appears only in the instructed probabilistic task):
  (1) $$y_{k}^{stria}=\varphi (w_{k}^{snc\_stria}*y^{snc}+\underset{i}{\sum }w_{i,k}^{in\_stria}*y_{i}^{in}+w^{pfc\_stria}*y^{pfc}$$
  where $y_{k}^{stria}$ is the output of the *k*th striatum neuron, $w_{k}^{snc\_stria}$ is a connection weight that equals 1 if the *k*th neuron stands for a Go signal or equals −1 if it stands for a NoGo signal, $y^{snc}$ is the output of the SNc neuron, $w_{i,k}^{snc\_stria}\;$ is a synaptic weight connecting the *k*th striatum neuron to the SNC, $y_{i}^{in}$ is the *i*th input of the network, $w_{i,k}^{in_{stria}}$ is a synaptic weight connecting the *k*th striatum neuron to the *i*th input of the network, $y^{pfc}$ is the output of the PFC/HC, $w^{pfc\_stria}$ is the synaptic weight connecting the appropriate striatum neuron to the PFC/HC (this weight has a value of 0.3 for all the simulations presented in the article) and φ (x) is the activation function, which form was chosen experimentally:
  (2) $$\varphi \left(x\right)=exp\;\left[-\alpha {\left(1-x\right)}^{2}\right]$$
  We used *a* = 8 for the simulations.

10. The activity of the GPe neurons is simply:
  (3) $$y_{k}^{gpe}=1-y_{k}^{striaNoGo}$$
  where $y_{k}^{gpe}$ is the output of the *k*th GPe neuron, and $y_{k}^{striaNoGo}$ is the output of the *k*th neuron of the D2 subsystem.
11. The following formula describes the activity of the GPi neurons:
  (4) $$y_{k}^{gpi}=max\left\{1-\frac{1}{2}y_{k}^{striaGo}-\frac{1}{2}\;y_{k}^{gpe},\;0\right\}$$
  where $y_{k}^{gpi}$ is the output of the *k*th GPi neuron, and *y^striaGo^* is the output of the *k*th neuron of the D1 subsystem.

Neurons must not produce negative outputs; therefore, the max function is applied.

12. The activity of the thalamus neurons is evaluated as:
  (5) $$y_{k}^{thalamus}=1-y_{k}^{go}$$
  where $y_{k}^{thalamus}$ is the output of the *k*th neuron of the thalamus layer
13. The activity of the PMC neurons is calculated in a few steps, due to the bidirectional connections between the PMC and thalamus:
  (6) $$x_{k}^{pmc}=\frac{1}{2}\;y_{k}^{thalamus}+\frac{1}{2}\underset{i}{\sum }w_{i,k}^{in\_pmc}*y_{i}^{in}+w^{pfc\_pmc}*y^{pfc}$$
  where $w_{i,k}^{in\_pmc}$ is the synaptic weight of connection between the *i*th input of the network and *k*th PMC neuron, and $w^{pfc\_pmc}$ is the synaptic weight of the connection between the PFC/HC, an appropriate neuron of the PMD with the value set to 0.05 for the computer simulations.
  If $\underset{k}{\max}\left\{x_{k}^{pmc}\right\}$ > 1, then normalization is applied:
    (7) $$x_{k}^{pmc}\leftarrow \;x_{k}^{pmc}/\underset{k}{\max}\left\{x_{k}^{pmc}\right\}$$
  Finally, the activation level of the neurons is evaluated as:
    (8) $$y_{k}^{pmc}=\varphi \left(x_{k}^{pmc}\right)$$
    where $y_{k}^{pmc}$ is the output of the *k*th PMC neuron.
14. The connection between the PMC and the striatum closes the signal-processing loop in the BG. We assumed that this connection is used to inform the striatum about the decision that was made by the network in order to direct the weight adaptation process that comes next. During the adaptation process, appropriate D1 and D2 neurons are exposed to the SNc stimulation.
15. The output of the network is the following:
  (9) $$y_{k}^{out}=1\;\mathrm{if}\;y_{k}^{pmc}=\underset{k}{\max}\left\{x_{k}^{pmc}\right\}\;\;y_{k}^{out}=0\;\mathrm{otherwise}$$

#### 2.2.2. Learning Algorithm in CGNN

The learning of the CGNN is based on ideas introduced in the Leabra framework [23]. Synaptic weights are tuned on the basis of the differences between the activities of neurons in two phases of the learning. Each learning trial is split into the minus phase and the plus phase. The former one is about the network making a decision (selection of stimulus among those provided to the input layer). The SNc activity is tonic. Further, the plus phase is applied, where calculations run in a similar way as during the minus phase, except for the SNc activity, which is altered. If a positive reward is received, the SNc responds with a DA burst. If the network receives a punishment as a consequence of the choice, the SNc outcome is a DA dip. All synaptic weights are modified according to the differences between the activities of neurons in both phases. Details of the learning algorithm are given below.

The initial values or synaptic weights are determined randomly. In the simulations, we used the Gaussian distribution with a mean value 0.05 and a variance of 0.01. In the minus phase, the network input is fed with a pair of symbols, and the responses of neurons in consecutive layers are evaluated according to the formulas provided before, assuming a tonic level of the SNc activity. In the simulations, we set all the synaptic weights. In the plus phase, the network receives feedback from the game. The calculations made in the previous step are performed once again but with a different level of the SNc activity. After a reward, the SNc provides the striatum with a DA burst, and this signal is sent only to the Go and NoGo neurons representing the stimulus that was just chosen. As a result, the Go neuron will be more active and NoGo neuron less active when the same stimulus is shown. After a punishment, the neurons of the D1 and D2 sublayers are exposed to a DA dip. In consequence, future presentations of the same stimulus will be followed by a higher excitation of the NoGo neuron and lower excitation of the Go neuron. The differences between the activity levels of the neurons in the plus and minus phases are evaluated in the following way:

(10) $$\begin{array}{c} ∆y_{k}^{stria}=y_{k}^{stria}\left(plus\right)-y_{k}^{stria}\left(minus\right)\\ ∆y_{k}^{pmc}=y_{k}^{pmc}\left(plus\right)-y_{k}^{pmc}\left(minus\right) \end{array}$$

Synaptic weights are updated according to the following formulas (resembling the delta rule known from the artificial neural networks literature):

(11) $$\begin{array}{c} w_{i,k}^{in\_stria}\leftarrow \;w_{i,k}^{in\_stria}+\alpha^{stria}*∆y_{k}^{stria}\\ w_{i,k}^{in\_pmc}\leftarrow \;w_{i,k}^{in\_pmc}+\alpha^{pmc}*∆y_{k}^{pmc} \end{array}$$

where *α^stria^* and *α^pmc^* are the learning rates of the striatum and PMC, respectively. The simulations presented here used 0.1 for both coefficients. It also assumed that the synaptic weights are nonnegative; therefore, if a weight update results in a negative value, it is simply replaced by 0.

Finally, the forgetting factor β is introduced:

(12) w←βw

We used β = 0.98 for the simulations.

#### 2.2.3. Parameters in CGNN

The CGNN parameters fall into three categories. The first category includes parameters encoding the network states: binary indicator of a stimulus appearing on the screen; the dopaminergic neurons activity (0 for dip, 0.5 for baseline and 1 for burst); the activity of neural units spanning from 0 (no activity) to 1 (the highest magnitude of excitation) and connection weights $w_{k}^{snc\_stria}\;$ between the SNc and the striatum that take a value of 1 for Go neurons and −1 for NoGo neurons. The choice of values of these parameters is mostly a matter of convention. In addition, the mathematical equations take a simpler form, and the computer code of the network is concise.

The second category are the design parameters, which values are crucial for the network to function in a proper way as a model in order to perform the reinforcement learning tasks. These are the following parameters used in the model: connection weights $w^{pfc\_stria}$ and $w^{pfc\_pmc}$ playing a crucial role in the Instructed Probabilistic Selection Task, coefficient a of the activation function, learning rates *α^stria^* and *α^pmc^* and the forgetting factor β. The interpretation of these parameters is rather straightforward. The weight $w^{pfc\_stria}\;$ indicates the strength of the influence of the instructions on the striatum activity expressed in the scale 〈0,1〉. More precisely, it tells how much gain striatum Go neurons receive from the PFC/HC. The weight $w^{pfc\_pmc}$ describes the influence of the instructions on the PMC in the Instructed Probabilistic Selection Task. The higher the value of $w^{pfc\_pmc},$ the more likely the PMC overrides the striatum with respect to decisions inconsistent with the instructions. The values of both weights are fixed to 0.3 in our simulation studies, because this particular value allows the network to unlearn the fake instruction in a number of trials that are similar to human subjects on this task.

The parameter a controls the shape of the activation function. We propose the activation function aimed at reducing the flexibility offered by the commonly used sigmoid function. The proposed function is also sigmoid-like; however, it maintains a similar shape for large values of the shaping coefficient a. From multiple simulations performed with different values of a, we found that its exact value does not matter much, because learnable weights can adjust themselves and achieve similar results regardless of the value of the a parameter. Therefore, we fixed it to the arbitrary value of 8. There is no point in trying to use it as a means to interpret behavioral data. The learning rates *α^stria^* and *α^pmc^* represent the strength of the reactions of the synaptic weights for the associated layers. We experimentally determined a value of 0.1 to reflect the learning speed of human subjects. The same values are used in reaction to punishments and rewards. The forgetting factor β may be easily mapped to the half-life of the memory trace. Our choice of 0.98 aimed to give limited memory of the stimulus quality that lasted for at least 20 trials, but in our study, every pair of stimuli reappeared after 7 trials at most. In summary, the model contains five design parameters that may be customized purposely. Our study used neural networks only to give a quantitative account of the behavioral data. Therefore, the same fixed values were sufficient to provide a neural network model behaving in a manner similar to human subjects.

The third category involves learnable interconnection weights: $w_{i,k}^{in\_stria}$ and $w_{i,k}^{in\_pmc}$. The total number of these weights is $3N^{2}$, where N is the number of stimuli to be learned. This is a low number in comparison to the hundreds or thousands of parameters of FGNN commonly used in the literature [19,20,22,23,24,26,45].

### 2.3. Reinforcement Learning Tasks

Let us consider a game where two stimuli are given to a subject. Here, let us assume that the stimuli are images shown to a subject on both sides of a computer screen. A subject is asked to choose between two stimuli appearing on both side of the screen. After the choice is made, a reward signal follows. The value of the reward depends on the stimuli chosen, and it is randomly drawn from the probability distribution assigned to a chosen stimulus. Two values define the distribution domain: one value to represent the positive reward and another one to represent the negative reward (punishment). The positive outcome is returned with probability *p_S_* and the negative outcome with probability 1 − *p_S_*, where *S* stands for stimulus. Each stimulus *S* is assigned a value of *p_S_*, and all those values that occur in the game are called contingencies. The key thing is that a subject playing the game does not know the contingencies and is expected to learn them during a trial-end-error process. During a single trial, a pair of stimuli is picked up and shown to the subject, the subject chooses one stimulus and immediately receives the reward (positive or negative).

In the Probabilistic Selection Task [22], a set of a few pairs of stimuli is prepared. Stimuli only appear in matched pairs, and the order of appearance is random. Additionally, stimuli in pairs appear on a randomly selected side of the screen. A subject aims to learn the contingencies in order to gain as much positive rewards as possible. A typical composition includes three pairs of stimuli: AB (80%/20%), CD (70%/30%) and EF (60%/40%). Notice that the first pair is the easiest to learn and the third pair the hardest. The Instructed Probabilistic Selection Task [43] is a version of the game above. The only difference is that the subject is misinformed that *B* is the best stimulus to choose or that *A* is the worse. The misleading cue is a part of the instruction manual read by the subject before the game starts. The Probabilistic Reversal Learning Task [44] is reduced to only a single pair of stimuli that is presented at each trial. The tricky thing is that, after several dozen trials, the contingencies are swapped (the reversal event). The subject is unaware of the moment of reversal, and despite this, he/she is expected to follow the contingencies.

The diameters of the neurons shown in Figure 1 are not randomly drawn. Let us perform one quick pass through the network to better grasp its functioning. Stimulus number one (A) and two (B) are presented to the network; hence, the two first neurons of the input layer are active. The SNc activity is tonic, as seen from the diameter of the only SNc unit. The striatum Go neuron representing stimulus A responds considerably stronger than its neighbor representing B. The opposite occurs in the NoGo sublayer. Figuratively speaking, in this network, the Go for the A command is supported by the NoGo for B. This logic is expressed by the GPi neurons. The neuron associated with stimulus A disinhibits the corresponding unit of the thalamus, allowing it to fire above the tonic level, whereas the B neuron keeps it close to the baseline. Then, the PFC/HC comes in and superimposes on the thalamus activity to finally drive the PMC toward the execution of the chosen B action. This is against the pattern of activity observed in the striatum. Notice that the PFC/HC already affected the striatum directly by pushing the activity of Go for stimulus B up a little bit. The output layer represents the final decision made by the network, which is to choose stimulus B.

## 3. Results

### 3.1. Simulation of Probabilistic Selection Task

To make the demonstrations of the network functioning readable, we worked with only one pair of stimuli, the first one having a 0.9 chance of being positively rewarded and the second one having a 0.2 reward rate. Therefore, at each trial, the input signal stayed the same. Note that this did not mean that the game screen stayed the same during whole game, because the side of the screen for the stimuli was randomly chosen. However, the stimuli must be visually recognized and classified, so the input layer expresses the same activity no matter the order of stimuli on the screen. These considerations did not matter here at all, because we directly activated the neurons of the input layer according to the pair of stimuli appearing at the current trial.

**Figure 2:**
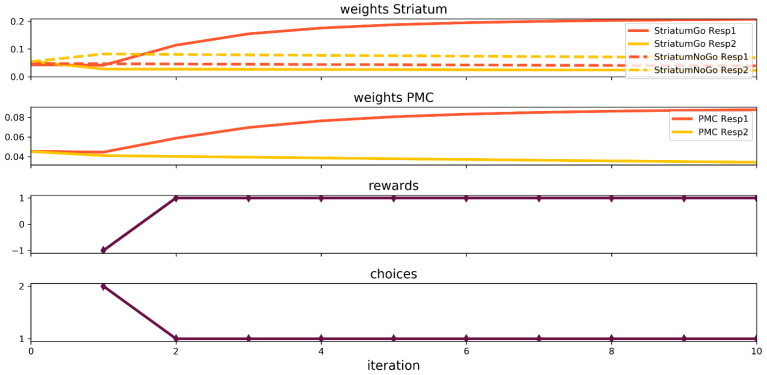
Neural network simulation of the Probabilistic Selection Task.

For this task, the PFC/HC is disconnected, since there is no instruction given. The network will go through 10 trials of the game. Stimulus number 1 is significantly better; therefore, the input–striatum and input–PMC weight connections of the neuron representing it will be followed and reported. The synaptic weight dynamics are illustrated in Figure 2. The zero trial is introduced to show their initial values. The initial random composition of the weights produced a network that prefers the second stimuli over the first. Only one punishment was enough to lean the network permanently toward stimuli number 1. Few further trials allowed the network to develop strong connections involving neurons representing the winning stimulus (red solid lines in Figure 2) in both the striatum and the PMC. These results are predictable and straightforward and are a good basis to grasp the neural network functioning mechanisms.

**Figure 3:**
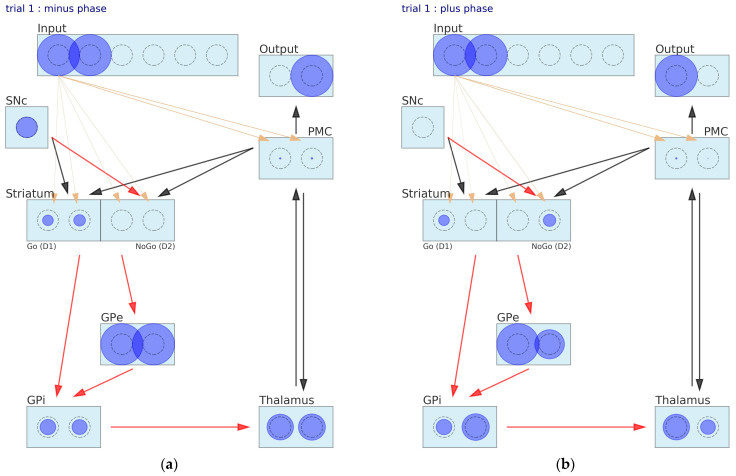

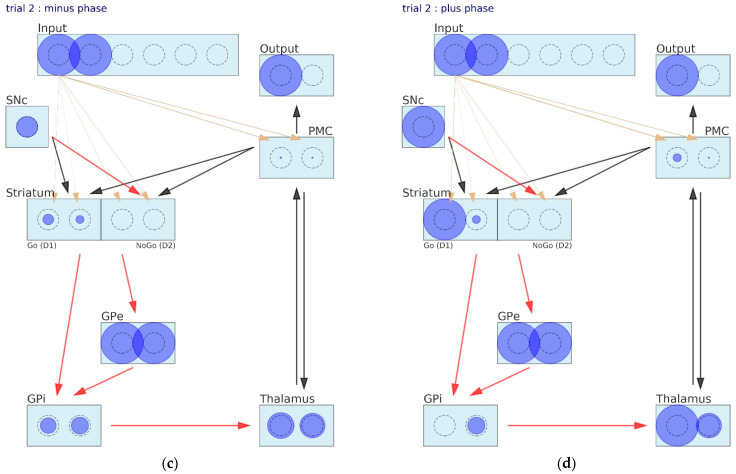
The neural network states during the Probabilistic Selection Task: (**a**) first trial—minus phase, (**b**) first trial—plus phase, (**c**) second trial—minus phase and (**d**) second trial—plus phase.

The network state at some trials is a collection of activities of all the neurons and values of the weights. The illustrations in Figure 3 provide visualization of the network state at the first and the second trials. At the minus phase of the first trial, the neuron activation was not significant, because it was calculated as a result of the initial random weights (Figure 3a). The network chose a worse stimulus and received a punishment. In consequence, the plus phase (Figure 3b) was performed in the presence of a DA dip, as seen from the empty rectangle representing the SNc. Unlike in the previous phase, the Go for the second stimulus unit of the D1 layer was turned off, and the activity of the NoGo for the second stimulus unit of the D2 layer went up. These changes of the activity pattern cause the thalamus to drive the PMC a little bit toward the choice of stimulus number 1. Still, the decision changed in favor only under the influence of the SNc reinforcement signal. After the first trial, the learning process takes place, and the synaptic weights adapt in response to the reinforcement signal. At the second trial, the pattern of activity is slightly different. The Go for one neuron is a bit more active than its neighbor, and the right choice is made in the minus phase (Figure 3c). After receiving the positive reward, the DA burst increased the differences even more (Figure 3d).

### 3.2. Simulation of Probabilistic Reversa Learningl Task

**Figure 4:**
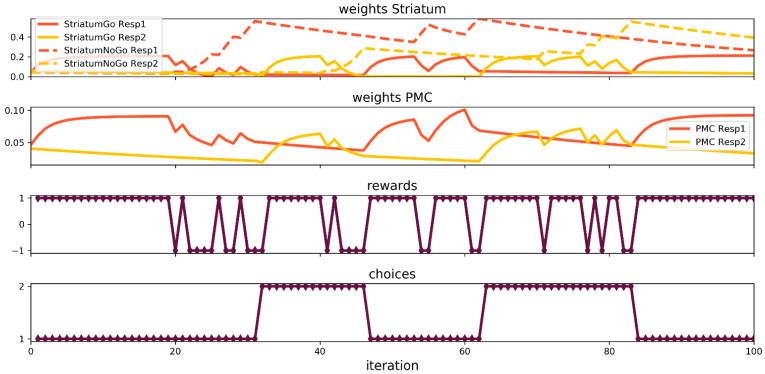
Neural network simulation of the Probabilistic Reversal Learning Task.

Initially, the contingencies were set up in the same way as in the probabilistic selection task and were kept unchanged during the first 20 trials. Then, there was a sudden reversal of contingencies; that is, the first stimulus became positively rewarded 20 percent of the time and the second stimulus 90 percent of the time. This set up was kept unchanged during the next 20 trials, followed by the swap of contingencies again until the end of the simulation at the 100th trial. At first, let us pay attention to the network behavior depicted at the lower part of the graph in Figure 4. Starting from the 20th trial, the number of punishments received by the network increased significantly, but the network changed the response to the second stimulus only after the next 10 trials passed. The next reversal event took place at the 40th trial and then, it took the network only seven trials to change its response to the first stimulus. Next, two reversal events required only a few trials of punishments to make the network change the response. An analysis of the course of the synaptic weights revealed that both the input–striatum and the input–PMC connections worked together to make the network solve the task. The input–PMC weights were alternately stronger and weaker. The input–striatum weights behaved similarly; however, the connections of the D2 units tended to be higher in magnitude (the domination of D2 over the striatum units might be the effect of random weights initialization). However, at first, the values of the weights must climb to a high enough level to be able to switch the network between responses, hence the relatively long time the network spends to change its mind after the first reversal events.

### 3.3. Simulation of Instructed Probabilistic Selection Task

**Figure 5:**
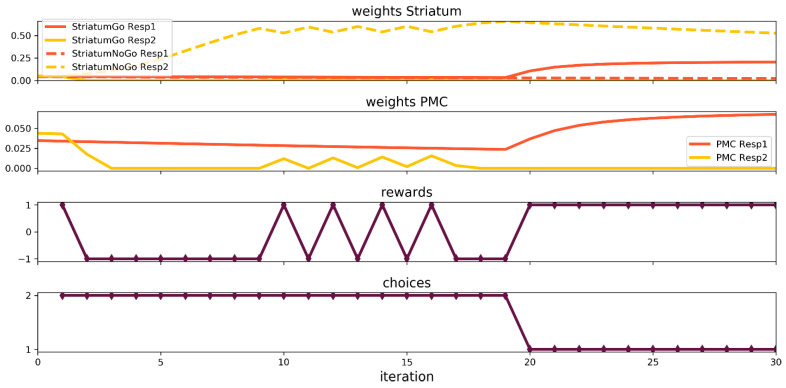
Neural network simulation of the Instructed Probabilistic Selection Task.

The instructed version of the game is the most interesting, because it requires additional nuclei operating during the simulation, namely the PFC/HC. The basic set up of the task is the same as in the probabilistic selection task, but the number of trials is raised to 30, and the effect of the instruction plays important role in the network functioning. The dynamics of the learning process is depicted in Figure 5. We can see that, despite a large number of punishment signals received by the network, it insists on choosing unprofitable stimuli for 19 trials. Starting from the 20th trial, the network begins to choose the first stimulus, acting against the misleading instructions. The connection between the input and the NoGo for the second stimulus plays a major role in overcoming the PFC/HC signal. However, when the unit of the PMC representing the first stimuli together with the Go for the first stimulus unit increased their connection strength, the network could switch to the second response.

**Figure 6:**
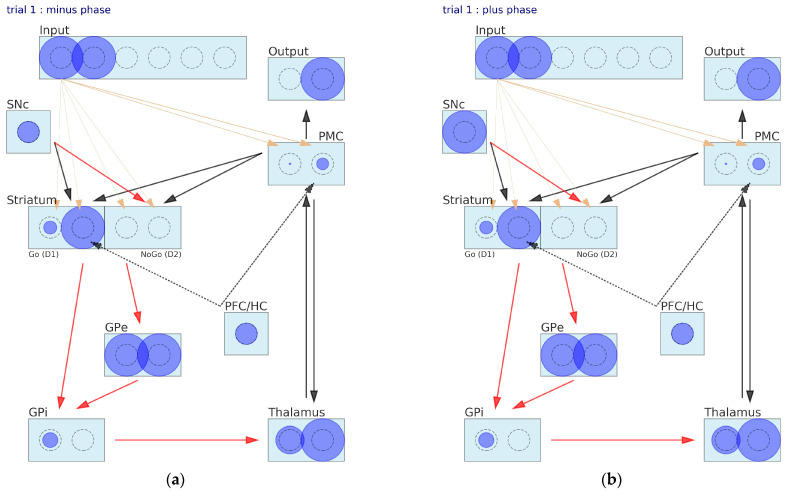

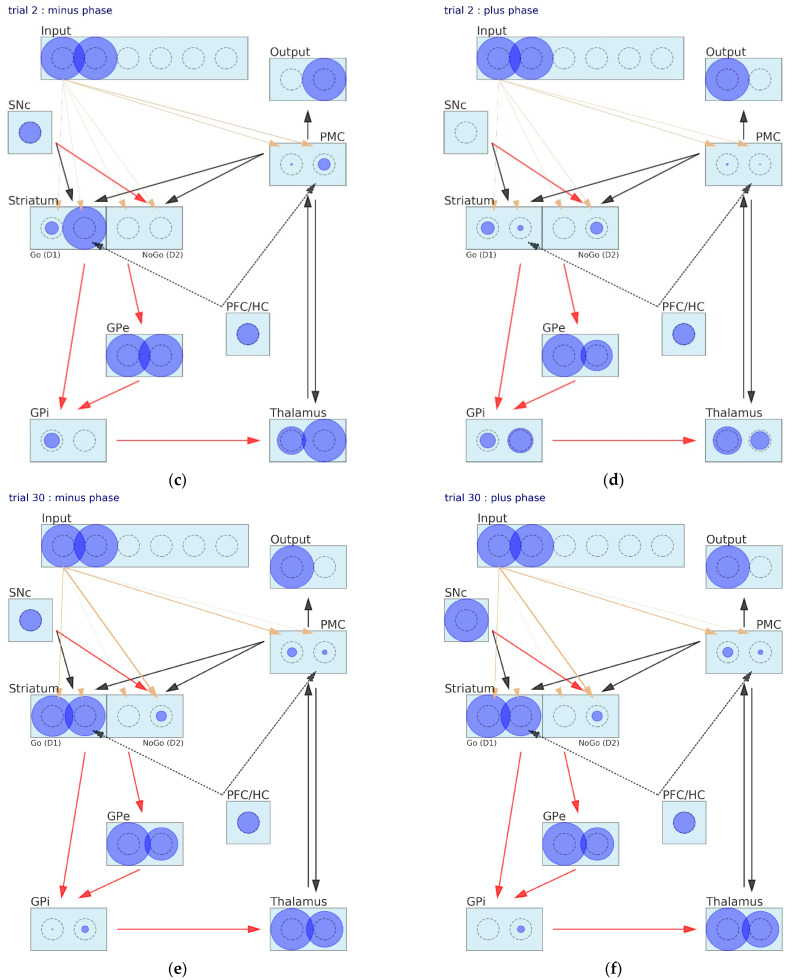
The neural network states during the Instructed Probabilistic Selection Task: (**a**) first trial—minus phase, (**b**) first trial—plus phase, (**c**) second trial—minus phase, (**d**) second trial—plus phase, (**e**) 30th trial—minus phase and (**f**) 30th trial—plus phase.

Now, let us take a look at the network states in the selected trials (Figure 6). At the beginning (Figure 6a), the PFC/HC acts to raise the activity of Go for the stimulus 2 neuron up to a high level, as well as the activity of the second unit of the PMC. Unfortunately, the rare reward event, having a 20% chance, took place, and the learning process went the wrong way. However, in the second trial, the same network response led to a punishment and, in consequence, to a DA dip (Figure 6d) in the plus phase. The striatum Go for stimulus 2 neuron activity was reduced significantly, and the NoGo for the stimulus 2 neuron woke up. The PMC layer activity remained low, despite extra stimulation by the PFC/HC. After 30 trials, the values of the synaptic weights were settled, and the final result could be seen in Figure 6e. Definitely, the strongest connection with the input layer developed the NoGo for the stimulus 2 unit of the striatum and the chosen stimulus 1 unit of the PMC. The Go for the stimulus 1 neuron of the striatum also gained a significant contribution to the signal flow. Interestingly, the level of activity of the NoGo for the stimulus 2 unit was moderate. It seemed that the strength of the synaptic connection did most of the job. Eventually, despite strong excitation delivered by the PFC/HC that encoded the instructions and biased the decision toward the worse stimulus, the NoGo for the instructed stimulus signal, together with the correct response of the PMC, overrode the misleading effects of the PFC/HC after going through the learning process.

## 4. Discussion

This work presents a new CGNN model of the BG derived from previous FGNN models [21,24,46] accounting for typical behavioral results from human studies on reinforcement learning tasks, such as the Probabilistic Selection Task [22], Instructed Probabilistic Selection Task [43] and Probabilistic Reversal Learning Task [44]. Although there is a natural tendency to design more detailed neural network models with an increasing number of neurons (with a notable exception described in Reference [46]), we decided to take a step in the opposite direction. The CGNN model preserves the fundamental anatomical structures present in the BG and reduces the complexity of the FGNN models to a great extent. Instead of modeling the functional units of the BG nuclei in a detailed manner, such as ensembles of a large number of biologically plausible neurons as in FGNN models, we proposed in CGNN to model the activity of entire groups of neurons by a simple mathematical equation (3) similar to the activation functions of single artificial neurons. This function may be considered as a representation of the average activity of a pool of neurons over time. Biological plausibility applies mainly to the connectivity and functionality of the CGNN layers, while individual units mimic the average behavior of real neurons. Simplification of the CGNN model reduces its flexibility but improves the readability of the signal flow and learning process in comparison to the FGNN models. The CGNN model allows to easily interpret the behavior of the network, since, in contrast to the FGNN models, it does not have many hard-coded parameters and an excess of adjustable synaptic weights. It is common in the literature to draw conclusions concerning the physiology of the learning process from a model with a huge number (tens of thousands) of adjustable weights [45,46].

Simple models, such as the presented CGNN, are of limited use in explaining complex biological phenomena, because they can account only for the most general properties of complex systems. In consequence, the CGNN design parameters, such as the values encoding DA bursts and dips, must be carefully determined for the research problem at hand. This is also the case for complex networks. A great example was given in Reference [47]; some symptoms of Parkinson’s disease related to kinetic disorders were simulated by modifying the model parameters, inspired by DA depletion. The variations in the model parameters altered the reaching movements, and the computer simulations reproduced the changes of the position and velocity observed in healthy and parkinsonian states. The main difference between the complex network and the CGNN is that the former is able to incorporate new situations into multiple adjustable parameters, whereas the latter must be carefully modified by the researcher. Thus, complex networks have some ability to compensate for their own design flaws, resulting from incomplete knowledge of the BG functioning. Compliance with the behavioral data improved, but the reasons behind this were at least partially hidden. Thus, the choice between more and less complex neural network architectures comes with consequences that should be taken into account in order for the model to answer specific research questions.

The simplified CGNN model is limited in the types of processes it can explain; however, those explanations are easily followed by human experts, and the conclusions drawn from its basis are more relevant. Illustrations of the internal CGNN states provided in our simulations demonstrated how easy it is to visually inspect the interplay between the input layer, the striatum and the PMC during synaptic weight adaptation in response to DA dips and bursts. Complex neural networks act as black boxes in this respect, whereas the CGNN may be treated as a logical machine that represents the logic behind the process in a simple and straightforward manner. However, the CGNN model should not be considered as a ready-to-use and complete solution for BG modeling. It is rather a proposition of the research direction aiming at providing a reference point for more thorough and complex models. In our opinion, there is a lack of BG models representing a more simple and thus readable approach that could serve as a starting point for novel hypothesis-driven research studies on humans, both in the general population as well as among patients with neurological and psychiatric disorders.

## 5. Future Directions

The CGNN was created with the aim to explain the behavior of healthy people, as well as neurological and psychiatric symptoms that arise from damages in the brain structures and/or disruptions in the connectivity between brain structures. Theory-based computational psychiatry and neurology provide a framework for the better comprehension, measurement and prediction of various phenomena, as well as for treatment development [48,49]. Neural network models are a useful tool enabling deeper insights into the computational signal processing that takes place once the model is given a cognitive task to learn. Inducing various constraints in the network structure and/or connectivity between various elements of the network architecture allows to observe changes in the network performance that might resemble symptoms typical for neurological or psychiatric disorders [2]. With time, new computerized tasks are being developed that are sensitive to the hypothesized neural computations that probe reward and punishment learning, cognitive control or reinforcement-based decision-making under uncertainty. Mathematical models can allow to provide quantitative estimates of individual performance parameters, yielding an assessment of the degree to which subjects rely on specific computations when learning and making decisions [18,50]. These parameters can be associated with the variation of the markers of neural activity (EEG and fMRI), genetics, pharmacotherapy, brain stimulation or illness symptomatology [51,52,53,54]. In the future, neural network and computational models could be beneficial in creating intervention protocols fostering a better focus on possible customized therapeutic approaches based on individual variations in BG dynamics. Moreover, the CGNN model presented in the article could be further developed to encompass cortico-subcortico-spinal connections that will allow creating rehabilitation programs improving sensorimotor retraining [55] or somatosensory restoration through brain–computer interfaces [56]. Additionally, the simplicity of the CGNN comes at the cost of the limited possibility to account for many phenomena that directly emerge from the behavior of dynamic neural units, such as, for example, temporal dynamics in cortico-subcortico-spinal excitability and their particular increased sensitivity in late-phase processing [57]. Thus, future BG neural network models might be further developed by including the dynamic neural network units while preserving the CGNN model qualities and advantages.

## 6. Conclusions

In our study, we proposed a CGNN model that preserves the fundamental anatomical structures present in the BG and reduces the complexity of the FGNN models, while it still has a functionality that mirrors the behavior of the most often used reinforcement learning tasks in human studies. The simplification of the CGNN model reduces its flexibility but improves the readability of the signal flow in comparison to more detailed FGNN models and, thus, can serve to a greater extent in the translation between clinical neuroscience and computational modeling.

## Data Availability

The data presented in this study are available on request from the corresponding author.
